# *De novo* mutation in the *ARHGAP32* gene endorses the implication of GTPase-activating proteins (RhoGAP family) in idiopathic autism spectrum disorder

**DOI:** 10.3389/fpsyt.2026.1754241

**Published:** 2026-02-06

**Authors:** Lara Cirnigliaro, Lucia Saccuzzo, Viviana Marzà, Martina Randazzo, Maria Perdichizzi, Corrado Romano, Marco Fichera, Renata Rizzo, Rita Barone

**Affiliations:** 1 Child Neurology and Psychiatry Unit, Department of Clinical and Experimental Medicine, University of Catania, Catania, Italy; 2 Medical Genetics, Department of Biomedical and Biotechnological Sciences, University of Catania, Catania, Italy; 3 Research Unit of Rare Diseases and Neurodevelopmental Disorders, Oasi Research Institute-IRCCS, Troina, Italy

**Keywords:** ARHGAP32, autism spectrum disorder, *de novo* mutation, jacobsen syndrome, rho GTPase activating proteins

## Abstract

**Introduction:**

ARHGAP32 gene (Rho GTPase Activating Protein 32) encodes a Rho GTPase activating protein, which is vital for the regulation of synaptic plasticity and cytoskeletal dynamics. ARHGAP32 (11q24.3) has been implicated as a candidate gene for Autism Spectrum Disorder (ASD) in Jacobsen syndrome, where a 243-kb terminal deletion encompasses its locus. A unique patient with *de novo* (DN) likely gene-disruptive mutation of *ARHGAP32* has been reported so far in the medical literature. The present study was undertaken to understand clinical, molecular, and neurobehavioral characteristics of ASD associated with a novel DN nonsense mutation in *ARHGAP32*.

**Methods:**

Clinical characterization included basal and follow-up assessment with standardized measures and comorbidities diagnosis. Trio exome sequencing analyses (WES) and variants annotation were performed.

**Results:**

WES analyses of a 6-year-old female patient with idiopathic ASD revealed DN heterozygous nonsense variant in *ARHGAP32* (NM_001378024.1: c.610C>T; NP_001364953.1: p.(Arg204Ter). The variant is predicted to introduce a premature stop codon, resulting in either a truncated protein or activation of nonsense-mediated mRNA decay, ultimately leading to loss of function. The patient presented with normative growth parameters and cranial measurements, with no congenital morphological anomalies. A diagnosis of idiopathic ASD was made at age 2. Developmental delays were observed, notably language regression beginning at 18 months, mild intellectual disability, and restricted interests accompanied by repetitive motor and verbal behaviors. Significant hyperactivity and attentional difficulties were observed. Over time, she exhibited borderline non-verbal cognitive functioning, persistent speech impairment, and was subsequently diagnosed with comorbid Attention Deficit Hyperactivity Disorder.

**Discussion:**

This study identifies shared neurobehavioral features of idiopathic Autism Spectrum Disorder (ASD) associated with *de novo* LoF mutations in ARHGAP32 and reinforces the involvement of RhoGAP family proteins in neurodevelopmental disorders. Taken together with previous evidence, our data support the role of *ARHGAP32* as a candidate gene for ASD, expanding the genetic spectrum.

## 1 Introduction

Autism Spectrum Disorder (ASD) is a neurodevelopmental disorder characterized by social communication impairment and restricted behaviours. Current prevalence worldwide is 1% with higher rates of 2.76% in the US and a male-to-female ratio of 3.8:1 (1, 2). ASD presents with a wide array of comorbidities including other neurodevelopmental disturbances such as ADHD, language disturbance, intellectual disability and motor coordination disorder all affecting independence and well-being (3, 4). Despite the great variability of symptoms severity, ASD outcome strongly relies on early identification by clinical screening tools based on developmental and behavioral features, prompting early intervention (5, 6).

Idiopathic ASD covers about 80% of diagnosed patients and it is believed to raise from the interaction between polygenic risk and environmental influences. ASD genetic architecture is complex, including gene mutations (*de novo* mutations (DNMs), rare inherited variants, and biallelic recessive variants), copy number variants (CNVs) and epigenetic modifications affecting the regulation of genes involved in neuronal development, synaptic structure and function, neurotransmission and electrophysiological activity, ultimately resulting in ASD development (7–10). DNMs define those mutations identified in the proband which are not present in the biological parents. Based on allele frequencies and natural selection principles, DNMs are considered to convey a stronger genetic effect so that the detection of DNMs in a proband may highlight novel ASD risk genes. In particular, DNMs caused by deleterious loss-of function (LOF) variants such as frameshift, splice site, and stop-gain deserved attention and are enriched in children with ASD and comorbid intellectual disability (11).

**Figure 1:**
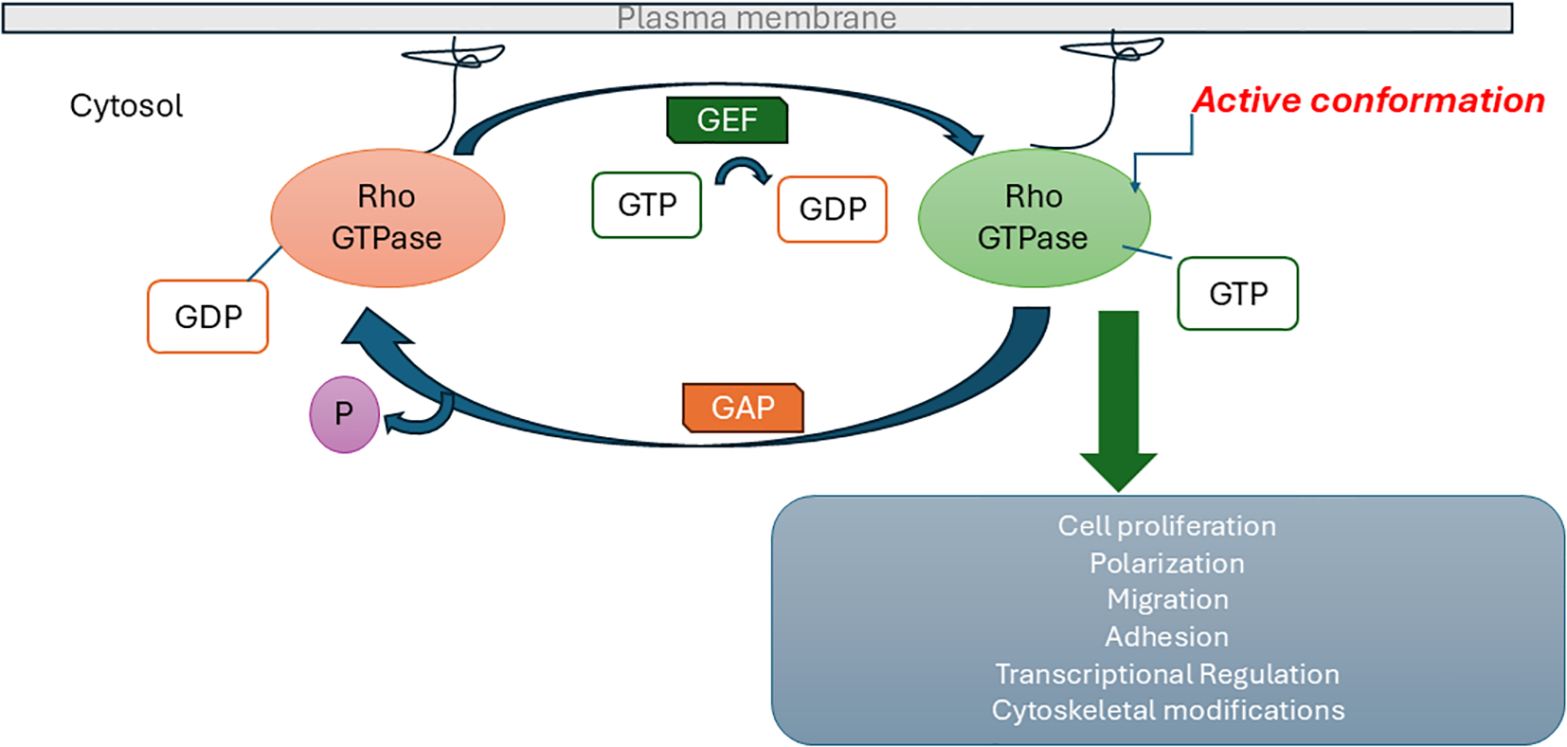
Regulation of Ras homolog (Rho) family GTPase signaling pathway by guanine nucleotide exchange factors (GEFs) and GTPase activating proteins (GAPs).

Numerous studies suggest that ASD risk genes may affect specific cellular mechanisms, such as chromatin remodeling, transcription, protein synthesis or degradation, or actin cytoskeleton dynamics, all processes ultimately regulating synaptic plasticity (12). Ras homolog (Rho) family GTPases, which belong to the Ras superfamily, are essential for the efficient completion of many physiological and developmental processes (13). They are regulated by GTPase activating proteins (GAPs) and guanine nucleotide exchange factors (GEFs). GEFs activate Rho by accelerating the intrinsic exchange of GDP for GTP and switch ON signal transduction. GAPs negatively regulate Rho by stimulating its slow intrinsic GTP hydrolysis activity and switch OFF signal transduction (**Figure 1**). GAPs are key regulators of the actin cytoskeleton that play critical roles in axonal outgrowth, dendritic spine morphogenesis and synapse formation (14, 15).

According with the Simons Foundation Autism Research Initiative (SFARI) database, it is evident that a number of ASD risk genes (2%) are involved in the Rho GTPase signaling pathway and almost 12% of GAPs have been proven to associate with ASD (16). Among GAPs, the ARHGAP32 (Rho GTPase Activating Protein 32), also known as RICS (RhoGAP Involved in the beta-Catenin-N-cadherin and NMDA receptor Signaling), is a neuron associated protein that regulates dendrite spine structure and strength, impacting postnatal remodeling of neural circuits during early brain development (17, 18). Evidence provided by the Human Protein Atlas indicates that ARHGAP32 expression is primarily restricted to excitatory neurons, inhibitory neurons, astrocytes, and oligodendrocyte precursor cells (19). Data from UniProt/SwissProt (20) indicate that mRNA expression of ARHGAP32 isoforms 1 and 2 is predominantly detected in the brain and testis. In addition, isoform 1 shows broader tissue distribution, with expression observed in the lung, liver, and spleen. The Tissue Expression Database (Jensen Lab, 21) documents that isoform 1 exhibits high levels of expression in the mammalian brain. Recent proteomic data showed that *ARHGAP32* levels were decreased in the dorsolateral prefrontal cortex of children with idiopathic ASD, whereas they were unaffected in ASD adults, thus supporting the role of *ARHGAP32* in postsynaptic density proteins that are required for the early development of neural circuits (22).

*ARHGAP32* (MIM*608541), located on chromosome 11q24.3, was proposed as a candidate gene for causing ASD in a subset of patients with 11q terminal deletion disorder (Jacobsen syndrome), which harboured a 243 kb deletion defining an autism critical region encompassing *ARHGAP32* (18). Drawing on this evidence, SFARI Gene designated ARHGAP32 as an ASD risk gene with a score of 2, indicating strong candidacy (23). To date, more than 20 inherited mutations in *ARHGAP32* have been identified in patients with ASD (24, 25).

Wang et al. (24) provided clinical data on the first patient identified with a *de novo* likely gene-disruptive mutation in the ARHGAP32 gene, whereas a *de novo* missense variant had been reported previously (26). The identification and phenotypic characterization of additional patients carrying mutations in the ARHGAP32 gene may further strengthen its association with ASD and provide greater insight into the related clinical features. In this context, we report the clinical, molecular, and neurobehavioral characteristics of a female ASD patient with a newly identified *de novo* nonsense mutation in ARHGAP32.

## 2 Materials and methods

### 2.1 Ethics considerations

This study was based solely on information and investigations that were carried out as part of the routine clinical care of ASD patients. All procedures performed in studies involving human participants were in accordance with the ethical standards of the institutional research committee at Policlinico “G. Rodolico-San Marco” Catania and with the 1964 Helsinki declaration and its later amendments. Written informed consent was signed by the parents of the proband.

### 2.2 Standardized measures

Psychomotor development and cognitive level were assessed according with age by measuring the general quotient (GQ) using the Griffiths Mental Development Scales, third edition (GMDS-III) (27) and the Nonverbal Intelligence Quotient (NVIQ) using the Leiter International Performance Scale, Third Edition (Leiter-3) (28). ASD symptoms were assessed using the gold standard tools for ASD diagnosis: Autism Diagnostic Interview—Revised (ADI-R) (29) and Autism Diagnostic Observation Program, 2nd edition (ADOS-2) (30). The Social Communication Questionnaire (SCQ), designed for detecting risk for ASD, was used to assess communication and social skills. A cut-off ≥11 has been shown to maximize sensitivity and specificity in younger children (31). Sensory processing difficulties, praxis and social participation were assessed using the Sensory Processing Measure (SPM) (32). The Repetitive Behaviours Scale – Revised (RBS-R) questionnaire completed by the parents was used in order to evaluate the spectrum of repetitive behaviours (33). The assessment of behavioural and emotional difficulties co-occurring with ASD was carried out using the Child Behavioural Checklist (CBCL) (ASEBA, Burlington, VT, USA). Abnormal T scores ≥ 60 were considered for internalizing, externalizing and total T scores. The Conner’s Parent and Teacher Rating Scale-Revised: short form (CPRS-R:S and CTRS-R:S) (MHS, New York, NY, USA) were used to assess inattention and hyperactivity problems and to exclude a possible comorbidity with ADHD.

### 2.3 Molecular analyses

Array Comparative Genomic Hybridization (aCGH) was conducted using the SurePrint G3 Custom CGH Microarray, 8x60K (Agilent Technologies, Inc.), following the manufacturer’s protocol, and employing Agilent sex-matched reference DNA (Euro female). Data extraction and analysis of the array were performed using CytoGenomics v.4.0.3 (Agilent Technologies, Inc.).

The nucleotide libraries required for exome sequencing (WES) were constructed using the SureSelect All Exon V6 kit (Agilent), following DNA extraction from peripheral blood. Sequencing of the DNA libraries was performed using next-generation sequencing (NGS) technology with 100-base paired-end reads using the NextSeq 2000 Illumina Sequencer (Illumina Inc.) according to the manufacturer’s instructions. The bioinformatics analysis involved aligning the sequences to the GRCh37/hg19 reference genome using the BWA-mem algorithm. Sequences exhibiting non-specific alignments were filtered out, and genetic variant calling was carried out using the GATK package, Samtools, and BCFtools to assess the coverage depth for the genes included in the requested genetic panel. The interpretation of the identified genetic variants was performed according to the guidelines set forth by the American College of Medical Genetics (34). Variant annotations were obtained by consulting several population databases (Exome Aggregation Consortium, 1000 Genomes Database, Exome Variant Server, gnomAD) as well as clinical genetic databases (OMIM, ClinVar, HGMD). Additionally, each variant was assessed for its potential impact on protein structure and/or function using tools such as PolyPhen2, SIFT, CADD, and MutationTaster, along with conservation profiles from PhastCons. Each potentially pathogenic variant was manually visualized using the Integrative Genomics Viewer (IGV) and technically confirmed by Sanger sequencing.

## 3 Results

### 3.1 Clinical report

The child is a 6-year-old female first-born to unrelated Italian parents. The father was 51 and the mother was 39 at the time of conception. She was delivered at 34 gestational weeks by caesarean section for placental abruption, after a pregnancy complicated by threats of preterm birth, treated with pharmacological therapy. Birth weight was 2320 g (50-75^th^ centile), length 48 cm (75-90^th^ centile) and head circumference 31.8 cm (50-75^th^ centile). At birth, she was admitted to the neonatal intensive care unit because of respiratory distress, treated with Continuous Positive Airway Pressure. She was also treated with phototherapy for neonatal jaundice. Brain ultrasound examination revealed moderate hyperechogenicity of the periventricular white matter.

Early developmental milestones were globally delayed with major concerns in social and language skills: she walked independently at 15 months; she had variegated babbling at 12 months with language regression from the age of 18 months. She was able to pronounce about four clear words at the age of 3 years and 6 months. Breast-feeding persisted until the age of 2 years and 6 months. Food selectivity was reported from the age of 18 months. At the age of 2 years, she attended the nursery school, showing a lack of interaction with the peer group. Full sphincter control was achieved at the age of 4 years.

At the age of 2 years, she was admitted to our unit for autism risk. Physical examination was unremarkable. The child showed reluctance to make eye contact during natural interactions and exhibited repetitive activities with mechanical objects. She had impaired social reciprocity and motor stereotypies, including hand flapping and turning around. There was no pointing or joint attention; imitative behaviour was limited, and pretend play was absent. Expressive language was characterised by producing vocalisations not always intended to communicate. Unusual sensory interests were also present (the child explored objects through smell and taste). Psychometric evaluation using the GMDS-III showed mild developmental delay (Chronological Age: 32 months; Developmental Quotient (DQ): 74, Developmental Age (DA): 15 months). Her developmental profile was heterogeneous, with lower scores on “language and communication” and “personal, social and emotional” subscales: (Foundations of Learning Scale: DQ:79, DA: 24 months; Language and Communication Scale: DQ:50; DA: 6 months; Eye and Hand Coordination Scale DQ:61; DA:20 months; Personal–Social–Emotional Scale: DQ 50; DA: 15 months; Gross Motor Scale DQ:66, DA: 22 months). She met criteria for autism on ADI-R subscales: qualitative abnormalities in reciprocal social interaction (score: 23; cut-off=10), and in communication (score: 14; cut-off=8) and restricted, repetitive, stereotyped patterns of behaviour (score: 9; cut-off=3). Standardized evaluation by ADOS-2 module 1 (Pre-Verbal/Single Words) showed a high level of symptoms typical for ASD diagnosis (Social Affect domain score: 18; Restricted repetitive domain score: 5; Total score: 23; ADOS-2 Comparison Score: 9, high level of ASD related symptoms). She started cognitive-behavioural therapy (CBT) and speech therapy from the age 2, with a progressive improvement in speech and social interaction over time.

Wake and sleep EEG recording, performed twice, at 20 months and 6 years respectively, yielded normal results. Repeated ophthalmological and audiological examinations were normal. Brain magnetic resonance imaging at study time (age 6) did not show any abnormal changes. Extensive metabolic screening including anion gap, lactic acid and blood ammonia levels, serum lysosomal enzymes analysis, serum transferrin glycoform analyses, urinary excretion of organic acids yielded normal results. In sum, the clinical and instrumental follow-up was consistent with a stable neurological course, with no evidence of progression over time.

Abnormalities involving growth, craniofacial features, ocular structures, cardiovascular system, respiratory and gastrointestinal tract, genitourinary system, skeletal system, skin and hair, and hematological profile were systematically assessed and excluded. In particular, cardiological evaluation, including ECG, did not reveal congenital heart defects, and no clinical, instrumental, or laboratory evidence of coagulopathies or other systemic disorders was identified.

**Figure 2:**
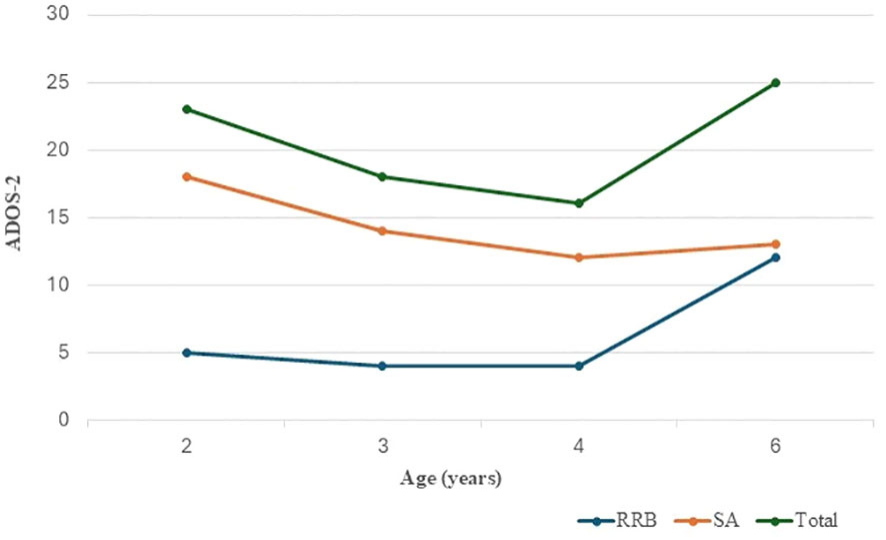
Clinical follow-up by ADOS-2 assessment: social affect (SA), restricted and repetitive behaviour (RRB) raw scores and ADOS-2 total score in relation to the study patient age. There is evidence of a reduction and stabilisation of the SA scores over time, indicating an improvement in the area of communication and reciprocal social interaction following CBT. An initial stabilisation of restricted and repetitive behaviours is also showed with a progressive worsening after 4 years of age, which influences the trend of the ADOS-2 total score.

At last follow-up visit (at the age of six), the weight was 19.8 kg (54th centile), the height 111 cm (45th centile) and the head circumference 51 cm (10-25th centile). The child showed hyperkinetic behaviour and motor and verbal stereotypies (echolalia). She was distractible and required re-direction. Expressive verbal language was characterised by producing vocalizations and single words. Formal assessment by the Leiter-3 indicated slightly lower than normal non-verbal cognitive profile (NVIQ: 76). Further evaluation by ADOS-2 confirmed the presence of a high level of symptoms relating to the ASD (**Figure 2**). The Social Communication Questionnaire (SCQ) was consistent with impaired communication and social skills (total score: 16). Assessment of sensory profile by SPM (Sensory Processing Measure) highlighted a definite dysfunction in the ‘social participation’, ‘vision’, ‘hearing’ and ‘planning and ideas’ subscales (SPM total T score: 80). The Repetitive Behaviours Scale – Revised (RBS-R) was consistent with the presence of restricted interests and repetitive behaviours, such as stereotyped hand and finger movements, turning around, jumping, rotating or lining up objects (RBS-R total score: 27). The Child Behaviour Checklist (CBCL) and the Conners Parent and Teacher Rating Scales: short form (CPRS-R:S) revealed high scores regarding attention problems and hyperactivity (T score: 84).

### 3.2 Genetic findings

**Figure 3:**
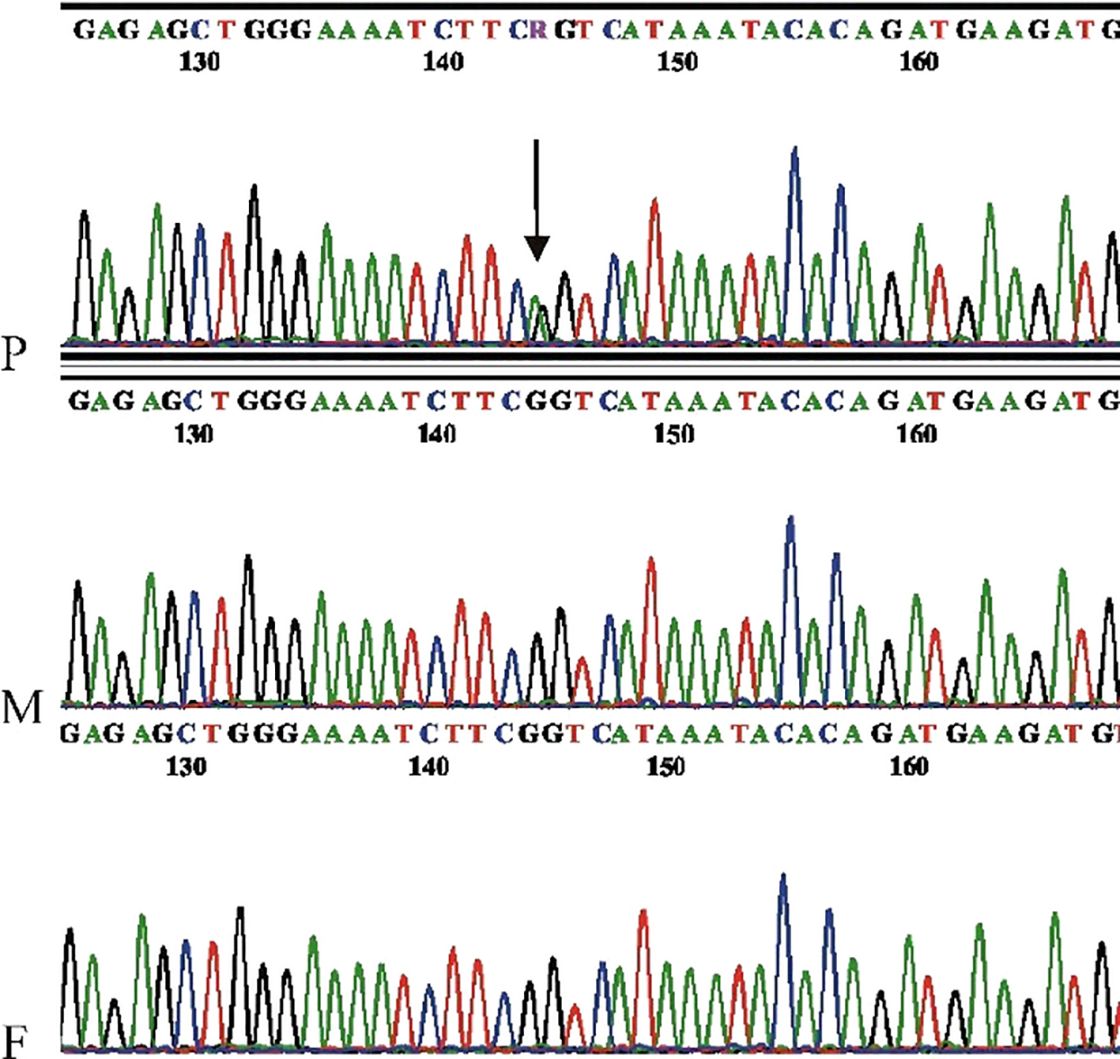
Electropherogram showing the ARHGAP32 c.610C>T variant. Sanger sequencing was performed in reverse orientation. The position of the mutation is indicated by an arrow. The electropherograms correspond to the proband (P), mother (M), and father (F), respectively. PCR amplification was performed using the following primers: forward primer (5′-GGCCTGATTCATAAGTATTTCC), reverse primer (5′-GAATCACCCTGCGTGTTCAGTA-3′).

Microarray analysis performed on the patient did not detect any clinically significant copy number variations (CNVs). Trio WES analysis revealed in the proband a *de novo* heterozygous variant in *ARHGAP32* [NM_001378024.1: c.610C>T, p.(Arg204Ter)], predicted to introduce a premature stop codon. The variant was confirmed by Sanger sequencing (**Figure 3**). This alteration likely results in either a truncated protein or triggers nonsense-mediated mRNA decay, leading to a loss of function (Lof). Notably, *ARHGAP32* has a probability of loss-of-function intolerance (pLI) score of 1, indicating extreme intolerance to loss-of-function variants, and the variant is extremely rare in population databases. Furthermore, the c.610C>T change lies in an exon (exon 7 out of 23, according to the canonical isoform) that is present in all annotated isoforms, suggesting that its deleterious effect impacts all expressed transcripts. According to ACMG criteria, this variant was classified as likely pathogenic (PVS1, PM2, PM6) and was predicted to be deleterious by the in silico prediction tool CADD (Phred score: 37), based on evidence from its *de novo* status, predicted inactivating effect, loss-of-function intolerance of the gene, and extremely low frequency in control populations. *ARHGAP32* Although *ARHGAP32* variants have been associated with autism risk (18, 25), the number of well-characterized ASD phenotypes remains limited (24), and the gene has not until now been classified as an OMIM disease gene.

## 4 Discussion

In the present study, we report on a 6-year-old female with a novel *de novo* nonsense variant in the *ARHGAP32* gene. The child had regular growth parameters and head circumference size and absence of facial dysmorphia and congenital morphological anomalies. She received a diagnosis of idiopathic ASD at the age of 2 years. She had mild developmental delay with language regression from the age of 18 months, mild intellectual disability, restricted interests and repetitive behaviours with motor and verbal stereotypies, marked hyperactive behaviour and attentional problems. Over the years she gained borderline non-verbal cognitive abilities, with impaired speech, and was diagnosed with comorbid attention deficit hyperactivity disorder (ADHD). WES revealed a c.610C>T, p.Arg204Ter *de novo* LoF variant in *ARHGAP32*, predicted to result in either a truncated protein or to induce nonsense-mediated mRNA decay, leading to a loss of functional *ARHGAP32* protein. ARHGAP32 has been suggested as a candidate gene contributing to ASD in a subset of individuals with terminal 11q deletion syndrome (Jacobsen syndrome) (18). The study patient did not show any CNVs and she underwent a comprehensive clinical screening for systemic comorbidities commonly associated with this syndrome, which yielded negative results. To our knowledge, this is the second report in which a *de novo* LoF of *ARHGAP32* is associated with full description of idiopathic ASD. Information of only one subject with ASD, a female patient with *de novo* likely gene-disruptive (LGD) mutation in *ARHGAP32* has been provided so far in the context of a large study on *de novo* genic mutations in a Chinese autism spectrum cohort (24). Based on the available data in the previously reported patient (24) and the current study, patients with *ARHGAP32 de novo* mutations and ASD were both females which presented with global developmental delay (motor and speech delay) and shared comorbidities, such as mild to borderline cognitive impairment and attentional problems (**Table 1**). They both had full blown autism phenotype with remarkable repetitive behaviour. They had neither epilepsy nor congenital morphological anomalies. Head circumference growth, physical features, EEG and brain MRI findings were normal in the present patient and unreported in the previous one (24). In sum, clinical evidence supports the role of RhoGAP family in neurodevelopmental disorders and endorses the role of *de novo* LoF mutations in *ARHGAP32* gene in idiopathic ASD. Further studies of patients with *de novo* mutations in this gene will be required to assess the significance of these observations.

**Table 1:** Demographic, clinical and molecular features of clinically characterized ASD patients with *ARHGAP32 de novo* variants.

Patients reference	P1 Wang et al., 2016	P2 This study
Sex	F	F
Age at diagnosis	nr	2 yrs
Age at study time	nr	6 yrs
Ethnicity	Asian	Caucasian
Consanguinity	nr	-
Genotype	NM_001378024.1: c.4755del NP_001364953.1: p.(Glu1586LysfsTer22)	NM_001378024.1: c.610C>T NP_001364953.1: p.(Arg204Ter)
CNV	nr	-
Inheritance	*de novo*	*de novo*
Clinical diagnosis	ASD - ID	ASD - ID
Neurological/Psychiatric features		
DD/ID	61 (IQ)	76 (NVIQ)
Microcephaly	nr	-
Macrocephaly	nr	-
Hypotonia	-	-
Speech delay	+	+
Regression	-	+
Seizures	-	-
Repetitive behaviour	+	+
Sleep problems	-	-
Hyperactive behaviour	-	+
Attention problems	+	+
Aggressive behaviour	-	-
Brain MRI	nr	-
Systemic features		
Facial dysmorphism	-	-
GI disturbances	+	-

−, absent; +, present; F, female; yrs, years; CNV, copy number variants; DD, developmental delay; ID, intellectual disability; IQ, intellectual quotient; NVIQ, non-verbal intellectual quotient; MRI, magnetic resonance imaging; GI, Gastrointestinal; nr, not reported.

Furthermore, the present study is consistent with the causative role of *de novo* LGD protein-truncating mutations in candidate ASD risk genes (including frameshift, stop-gain, splice-donor, and splice-acceptor). These were found two-fold more common in ASD patients, particularly in the presence of intellectual disability, than in their unaffected siblings (9), contributing significantly to ASD etiology and phenotypic spectrum (7). A landmark study using exome analyses to investigate rare coding variations in 3,871 autism cases and 9,937 ancestry-matched or parental controls, found that autosomal genes strongly associated with autism risk harbour *de novo* LoF mutations in over 5% of autistic subjects and encode proteins implicated in synaptic function, chromatin modeling and transcriptional regulations (26).

*ARHGAP32* encodes a Rho GTPase activating protein that is essential for the regulation of synaptic plasticity and cytoskeletal dynamics and influences the establishment and maintenance of functional neural circuits by modulating the Rho GTPase signalling pathway. Several lines of evidence have suggested that deficiency of the GTPase-activating protein encoded by *ARHGAP32* in neurons and dysfunction of Rho GTPase signalling contribute to the pathogenesis of ASD and may be associated with the autistic phenotype (14). Studies in animal models have shown reduced γ-aminobutyric acid type A receptor (GABA_A_R) levels in *ARHGAP32*-deficient neurons with impaired GABA_A_R -mediated synaptic transmission (35), further highlighting the importance of *ARHGAP32* in synaptic function. The longer spliced *ARHGAP32* isoform, PX-RICS, has also been shown to play a critical role in emotional learning in the amygdala by modulating GABAergic synaptic plasticity. Thus, impairment of the PX-RICS-dependent GABA_A_R transport mechanism leads to atypical social-emotional processing, resulting in autistic-like social behaviour in mouse models (36).

In conclusion, we identified a novel *de novo* LoF mutation in the *ARHGAP32* gene in a female patient with ASD, and highlighted overlapping clinical features with a previously reported *de novo* mutation in *ARHGAP32*, including its occurrence in females with ASD and shared comorbidities such as mild-to-borderline intellectual disability and attention deficit. Additional studies are required to confirm the causative role of *ARHGAP32* gene in the genetic etiology of ASD. DNMs are most likely to have a potential genetic risk effect, so there is considerable interest in detecting novel DNMs supporting ASD risk gene identification. Taken together with previous evidence, our data support the role of *ARHGAP32* as a candidate gene for ASD, expanding the genetic spectrum.

## Data Availability

The original contributions presented in the study are included in the article/supplementary material, further inquiries can be directed to the corresponding author/s.
